# Haplotype sharing correlation of alcohol dependence on chromosomes 1–6 in 93 nuclear families

**DOI:** 10.1186/1471-2156-6-S1-S79

**Published:** 2005-12-30

**Authors:** Dajun Qian

**Affiliations:** 1 Department of Biostatistics, City of Hope National Medical Center, 1500 E. Duarte Road, Duarte, California 91010, USA

## Abstract

Haplotype data contain signatures of ancestral alleles and increased information for mapping genes associated with complex traits. The motivation of this paper is to test the feasibility of a recently developed haplotype reconstruction algorithm and to perform haplotype-sharing correlation (HSC) analysis in nuclear families using data provided by the Genetic Analysis Workshop 14 and the Collaborative Study of the Genetics of Alcoholism. As an exemplary analysis, haplotype data on chromosomes 1–6 were reconstructed from genotype data in 93 nuclear families by minimizing both the recombinants in within-family haplotypes and the tree distance in between-family haplotypes. HSC analysis was performed using the best set of reconstructed haplotypes, and chromosome-wide significance was evaluated using a permutation procedure. Three markers were found to have significant haplotype associations with DSM-IV alcohol dependence that exceeded the 0.05 level of chromosome-wide significance: marker rs895941 at 36.7 cM on chromosome 3 (*p *= 0.03), marker rs1631833 at 109.1 cM on chromosome 4 (*p *= 0.008), and marker rs953887 at 74.2 cM on chromosome 6 (*p *= 0.02). These results indicated the usefulness of HSC analysis and provided further evidence on chromosome regions associated with alcohol dependence.

## Background

Haplotype data on dense markers contain local linkage disequilibrium information on historical recombination and mutation events, and the knowledge of haplotype structure has lead to a growing belief that haplotypes may hold the key to understanding and identifying genetic variants underlying complex traits [1]. The availability of thousands or even millions of single nucleotide polymorphisms (SNPs) on the human genome requires systematic analysis in coping with both optimal modeling and computational efficiency. Haplotype sharing methods have shown promising results in gene mapping analyses in complex settings [2-6]. To analyze the SNP data provided by the Collaborative Study of the Genetics of Alcoholism (COGA), we implemented an algorithm for haplotype reconstruction under the criteria of minimum recombinants and coalescent tree, and performed haplotype-based association analysis by the haplotype-sharing correlation (HSC) method [6,7]. The purpose of this paper is to evaluate the feasibility of our haplotype reconstruction algorithm and the HSC method when applied to nuclear family data with a limited amount of missing genotypes.

## Methods

### Data

The original COGA data contained 143 families, with an average family size of 11.2 ± 5.4 members and 9.3 ± 4.3 of them having SNP genotype data. To evaluate the feasibility of haplotype reconstruction and HSC analysis, we chose to analyze a dataset on chromosomes 1–6 in all 93 nuclear families with genotype data for both parents and at least 3 offspring. These nuclear families had an average family size of 6.6 ± 1.7 (range from 4 to 14), and contained a low proportion of 0.1% missing SNP genotypes. The phenotype variable to be analyzed was DSM-IV alcohol dependence, which was coded as ordered values of 1 for "pure unaffected", 2 for never drank, 3 for unaffected with some symptoms, and 5 for affected, and was treated as a continuous variable in HSC analysis.

### Haplotype reconstruction

Haplotypes in nuclear families were reconstructed in 2 steps using a search algorithm under the criteria of minimum recombinants and coalescent tree. In step 1, all possible minimum recombinant haplotype configurations (MRHCs) were reconstructed within each family under the criteria of minimum recombinants [8]. The number of possible MRHCs in each family depends on both the family size and the transmission process of haplotypes, and some nuclear families may have more than 100 MRHCs that are consistent with the observed genotype data.

In step 2, each MRHC in each nuclear family was evaluated by fitting the combination of its founder haplotypes and all founder haplotypes in other families to a coalescent tree structure, where the founder haplotypes were referred to the 4 parental haplotypes in each family. The MRHC corresponding to a coalescent tree with minimum tree distance was selected as the optimal solution of haplotypes. The computation of tree distance in a set of haplotypes is as follows. First, the sharing in each pair of haplotypes is quantified as the number of identical-by-state intervals summed over all markers, and the distance between 2 haplotypes is defined as the observed sharing subtracted from the maximum possible sharing. Second, a single haplotype showing the minimum sum distance with all other haplotypes is chosen as the ancestral haplotype. And third, all haplotypes are connected one-by-one starting from the ancestral haplotype using a minimum spanning tree algorithm [9], and the tree distance is defined as the minimum distance that connects all the haplotypes.

### Haplotype-sharing correlation

The HSC method evaluates the correlation between phenotype similarity and haplotype sharing at each marker *m *in all pairs of pedigree founder haplotypes [6,7]. The HSC statistic can be written as



where



Both phenotype similarity *Y*_*kl,m *_and haplotype similarity *X*_*kl,m *_are calculated for each pair of pedigree founder haplotypes *k *and *l *at marker *m*, and  and  are the average values of *Y*_*kl,m *_and *X*_*kl,m*_, respectively, in all *N *pairs of haplotypes *k *<*l*. Specifically, a) the phenotype similarity *Y*_*kl,m *_= *y*_*km*_*y*_*lm*_, where *y*_*km *_(or *y*_*lm*_) is the within-family phenotype score for haplotypes *k *at marker *m*, and is quantified by the sum of phenotype residuals in family members carrying the marker *m *of haplotype *k *identically by descent; b) the haplotype similarity *X*_*kl,m *_is quantified by the number of intervals shared identical by state surrounding marker *m*; and c) *r*_*m *_is the Pearson correlation coefficient of *Y*_*kl,m *_and *X*_*kl,m *_in all *N *pairs of pedigree founder haplotypes, and *z*_*m *_is the Fisher's transformation of *r*_*m*_. If *H *is the number of pedigree founder haplotypes in all unrelated families, then the number of all pairs of pedigree founder haplotypes is *N *= *H*(*H *- 1)/2. The significance of HSC statistic *z*_*m *_is evaluated at each marker by a permutation procedure for chromosome-wide significance [7].

## Results

On average, we were able to reconstruct haplotypes at all markers on a whole chromosome in 98.2% of the nuclear families. For the other 1.8%, haplotype phases on less than 1% loci could not be inferred with uncertainty conditional on the criterion of minimum recombinants, and those loci were treated as missing in reconstructed haplotypes. A haplotype at a missing locus was considered to have no sharing with any other non-missing haplotypes.

**Figure 1:**
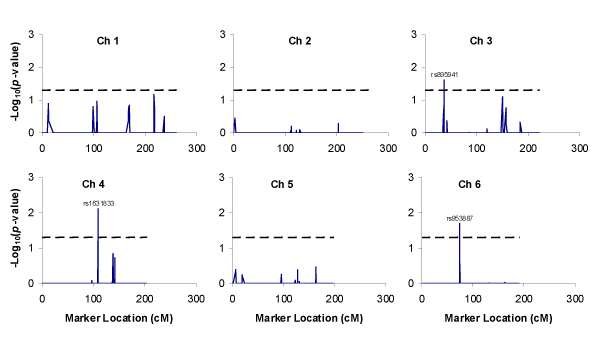
**HSC analysis of DSM4 alcohol dependence on chromosomes 1–6 in 93 nuclear family**. Horizontal dashed line is the threshold of chromosome-wide 0.05 significance level with line length equals to chromosome length, and three markers with statistical significances exceeding the threshold are labeled with their marker names.

In an HSC analysis on chromosomes 1–6 in 93 nuclear families, three markers on chromosomes 3, 4, and 6, respectively, were found to have significant associations with DSM-IV alcohol dependence that exceeded the 0.05 level of chromosome-wide significance (Fig. 1). Marker rs1631833 at 109.1 cM on chromosome 4 was found to have the strongest haplotype association among the 6 chromosomes analyzed (*p *= 0.008). Marker rs895941 at 36.7 cM on chromosome 3 and marker rs953887 at 74.2 cM on chromosome 6 were the other two markers revealed significant haplotype association (*p *= 0.03 and *p *= 0.02, respectively).

## Discussion

We have developed a 2-step algorithm for haplotype reconstruction in nuclear families that avoids the assumption of linkage equilibrium by minimizing the recombinants in within-family haplotype transmissions and fitting all parental haplotypes under a coalescent tree structure. The choice of analyzing nuclear families each with a large number of offspring was mainly under the feasibility consideration for testing the algorithm. When SNP data on chromosomes 1–6 were analyzed, haplotypes on less than 0.1% a loci in 1.8% of nuclear families could not be inferred with certainty. One possible reason for the failure of haplotype reconstruction in some nuclear families is the uncertainty in counting the number of recombinants in the presence of missing genotypes. We are currently investigating the failures and alternative approaches in order to improve the haplotyping performance in the presence of missing genotypes.

The HSC method evaluates the correlation between phenotype similarity and haplotype sharing at each study marker in all pairs of pedigree founder haplotypes. When applied to the COGA data on chromosomes 1–6, 3 markers were found to have significant haplotype associations with DSM-IV alcohol dependence. The most significant signal at 109.1 cM on chromosome 4 was consistent with the strong linkage signal found on the same region using the maximum number of drinks ever consumed in a 24-hour period as an alcoholism phenotype [10]. On a different note, the HSC method is not designed for controlling population stratification, although empirical results have indicated its robustness against allele heterogeneity when compared to allelic and haplotypic family-based association test [7]. Additionally, the HSC analysis does not consider within-family phenotypic correlations, and such a treatment may have an adverse effect in detecting the true associations.

Both the haplotype reconstruction and the HSC methods employed in this study have potential applications for haplotype-association studies under settings of both family-based and case-control designs. To improve the mapping of susceptibility regions associated with complex traits, clustering approaches, such as described by Yu et al. [11], may be employed in both haplotype reconstruction and haplotype association analyses. With clustering analysis, the plausibility of a candidate haplotype pair will be evaluated not by all existing haplotypes but only those believed to have the same ancestral origin. By the same token, clustering analysis will also increase the power of association analysis by reducing the ancestral heterogeneity in haplotypes associated with the same or similar phenotypes. We believe the HSC approach employed in this article, and its modified version to incorporate data adaptive clusters, will be useful in the mapping of complex traits.

## Abbreviations

COGA: Collaborative Study of the Genetics of Alcoholism

GAW14: Genetic Analysis Workshop 14

HSC: Haplotype sharing correlation

MRHCs: Minimum recombinant haplotype configurations

SNP: Single-nucleotide polymorphisms
